# Hollow Mesoporous Carbon Nanospheres Derived from Metal–Organic Frameworks for Efficient Sono-immunotherapy against Pancreatic Cancer

**DOI:** 10.34133/cbsystems.0247

**Published:** 2025-05-09

**Authors:** Libin Chen, Haiwei Li, Jing Liu, Yunzhong Wang, Shengmin Zhang

**Affiliations:** ^1^ Liaoning Cancer Hospital & Institute, Cancer Hospital of Dalian University of Technology, Cancer Hospital of China Medical University, Shenyang 110042, China.; ^2^ Department of Ultrasound Medicine, The First Affiliated Hospital of Ningbo University, Ningbo 315010, China.; ^3^ Department of Radiology, The First Hospital of China Medical University, Shenyang 110001, China.

## Abstract

Sono-immunotherapy is expected to effectively enhance treatment efficacy and reduce mortality in patients with pancreatic cancer. Hence, efficient applicable sono-immunotherapy systems are urgently needed for the treatment of this condition. In this study, hollow mesoporous carbon (HMC) nanoparticles were prepared using the sacrificial template method. These nanoparticles had a porphyrin-like structure and could generate singlet oxygen more efficiently than commercial TiO_2_. Cellular assays showed that HMC killed tumor cells in the presence of ultrasonication, primarily by inducing apoptosis. HMC could also accelerate the release of immune factors by tumor cells, thereby activating dendritic cells and enhancing the efficacy of immunotherapy. Experiments in tumor-bearing mice and in situ pancreatic cancer tests showed that HMC, in combination with the small-molecule inhibitors of programmed cell death ligand 1, could reduce tumor growth via the generation of reactive oxygen species following ultrasonication. HMC could enhance the efficacy of immunotherapy by disrupting the immunosuppressive tumor microenvironment and promoting the accumulation of immune cells. Accordingly, in vivo sono-immunotherapy was achieved, and the growth of transplanted tumors and in situ tumors could be reduced. In conclusion, this study proposes a novel method for the preparation of HMC nanoparticles and demonstrates their potential in tumor treatment. Additionally, owing to their unique structure, these HMC nanoparticles could be used for different combination therapies tailored based on specific clinical requirements.

## Introduction

Pancreatic cancer is a malignancy associated with a high rate of mortality [1–3]. Despite important advancements in medical research and technology, the treatment of pancreatic cancer remains challenging [4–6]. Current chemotherapeutic agents often demonstrate low efficacy against pancreatic cancer cells, and patients frequently experience poor tolerance to these drugs [7–9]. Additionally, the limitations of radiotherapy prevent its complete adoption in a wide range of patients. Although conventional therapies can offer some benefits when used in combination with immunotherapy, the complex microenvironment of pancreatic cancer allows tumor cells to suppress the immune response through the secretion of specific molecules [10–15]. Hence, novel therapeutic approaches are urgently warranted. Recent studies have shown that ultrasonic treatment can temporarily disrupt the barriers (such as fibrous tissue) around pancreatic tumors, enhancing drug delivery to the tumor and eliciting an immune response that improves treatment outcomes [16–20]. Hence, the application of sonosensitizers with effective sonodynamic properties, in combination with immunotherapy, could be expected to substantially improve treatment efficacy in patients with pancreatic cancer and limit the damage caused by this condition [21–25].

Recently, metal–organic framework (MOF)-derived carbon materials have garnered considerable interest among researchers owing to their unique structures and exceptional performance [26–28]. These materials have found widespread applications in energy storage [29], medical imaging [30], and cancer therapy [31,32]. Compared to conventional carbon-encapsulated materials, MOF-derived carbon materials offer several advantages, including more uniform channels, larger specific surface areas, and more regular metal-oxide structures [33–36]. These features make them particularly valuable in the field of biomedicine [37,38]. For example, Pan et al. [39] utilized carbon nanoparticles fabricated by calcining zeolitic imidazolate frameworks (ZIFs) for efficient sonodynamic therapy. Similarly, Jiang et al. [40] developed regular nanoparticles via MOF calcination to enhance resolution in magnetic particle imaging. These studies demonstrate the substantial research and diverse application potential of MOF-derived carbon materials in biomedicine [41,42]. However, there is limited research on the synthesis of MOF-derived hollow carbon nanomaterials and their specific biomedical applications.

**Fig. 1.**
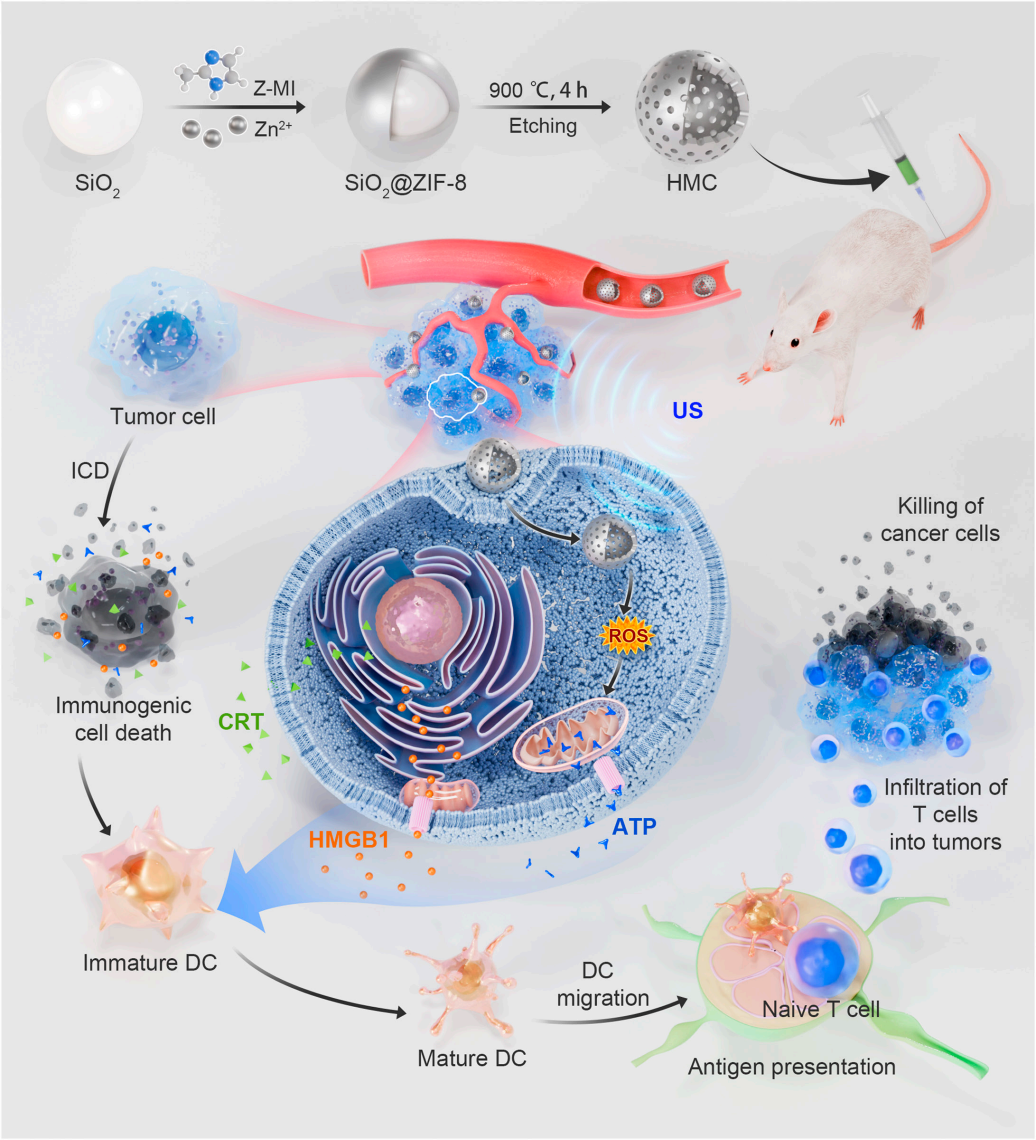
Preparation routine of hollow mesoporous carbon (HMC) and schematic of tumor growth inhibition. After tail vein injection, HMC accumulated in tumor tissues, promoting the production of reactive oxygen species (ROS) under ultrasound treatment. ROS induced tumor cell death, leading to the release of high mobility group protein B1 (HMBG1) and calreticulin (CRT) and the consequent activation of dendritic cells (DCs). This enabled T cells to further eliminate tumor cells. Z-MI, 2-methylimidazole; ICD, immunogenic cell death; ZIF, zeolitic imidazolate framework; US, ultrasound; ATP, adenosine triphosphate.

In this study, we propose a novel method to fabricate hollow mesoporous carbon (HMC) nanoparticles through calcination-etching using SiO_2_@ZIF-8 as a sacrificial template (Fig. 1). With its porphyrin-like structure, these HMC nanoparticles could convert oxygen into singlet oxygen in the presence of ultrasound (US) treatment, offering a stronger conversion ability than commercial TiO_2_. In vitro cellular assays demonstrated that in response to US, HMC nanoparticles could effectively enter cells and produce reactive oxygen species (ROS), leading to cell death. Cell death, in turn, promoted the release of high mobility group protein B1 (HMBG1) and calreticulin (CRT) from tumor cells, thus promoting the maturation of dendritic cells (DCs). Finally, experiments in animal models demonstrated that HMC nanoparticles had good biocompatibility, could accumulate in tumors after tail vein injection, and could inhibit tumor growth following US treatment. These nanoparticles could be loaded with small-molecule inhibitors of programmed cell death ligand 1 (PD-L1), promoting their accumulation on the surface of tumor cells and enhancing the immune response, thereby achieving sono-immunotherapy. Experiments in an in situ tumor model of pancreatic cancer further confirmed that this strategy enables the effective treatment of in situ pancreatic cancer.

## Methods

### Synthesis of HMC

First, we dispersed 100 mg of 120-nm SiO_2_ in 20 ml of methanol and stirred the solution until it was uniform. Next, we added 150 mg of zinc nitrate hexahydrate to the solution and continued stirring for 4 h before adding 250 mg of 2-methylimidazole. This was allowed to age at room temperature for 20 h. Subsequently, the product was collected by high-speed centrifugation and washed 3 times with methanol and ethanol. It was then dried in a vacuum oven to obtain SiO_2_@ZIF-8. We then dispersed 100 mg of SiO_2_@ZIF-8 in a porcelain boat and placed it in a tube furnace, heating it at a rate of 5 °C/min to 900 °C and maintaining its temperature for 4 h and then cooling it to room temperature. The resultant powder was dispersed in an ammonia solution to remove SiO_2_. After centrifuging the mixture, the product was washed 3 times with deionized water and ethanol. Finally, the product was dispersed in deionized water and freeze-dried to obtain a black powder.

### Cell culture

PAN02 cells were cultured in complete high-glucose Dulbecco’s modified Eagle medium. The medium was supplemented with 10% fetal bovine serum (FBS) and 1% penicillin–streptomycin. The cells were maintained in a 37 °C incubator with 5% CO_2_.

### Statistical analysis

The presented statistical data are expressed as mean ± standard deviation. The calculated *P* values were determined through a Student *t* test (***P < 0.001; **P < 0.01; *P < 0.05).

## Results and Discussion

### Preparation and testing of HMC

**Fig. 2.**
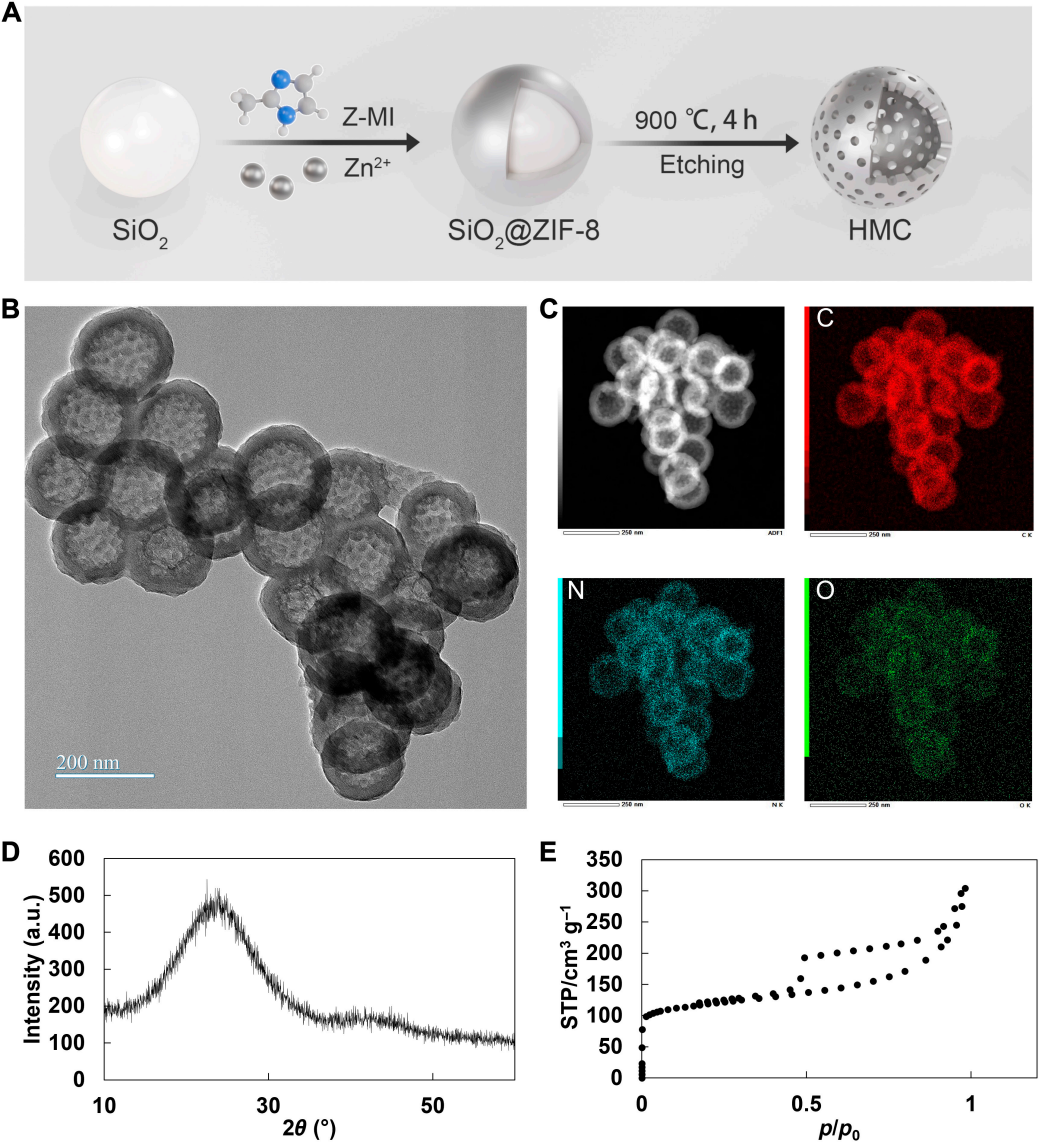
(A) Preparation routine of HMC. (B) Transmission electron microscopy image of HMC. (C) Elemental mapping of HMC. (D) X-ray diffraction pattern of HMC. (E) Brunauer–Emmett–Teller (BET) analysis of HMC. STP, standard temperature and pressure.

Herein, the MOF-derived HMC nanospheres were prepared using the method shown in Fig. 2A. First, a layer of ZIF-8 was grown on the surface of SiO_2_ to obtain SiO_2_@ZIF-8, which was subsequently used as the sacrificial template (Figs. S1 and S2). Then, SiO_2_@ZIF-8 was calcinated at 900 °C for 4 h to carbonize ZIF and remove zinc ions. The calcined products were collected and reacted with ammonia to remove the SiO_2_ template. Then, after several centrifugal washes, HMC nanospheres were obtained.

The structure of the as-prepared HMC nanoparticles was tested. Transmission electron microscopy images showed that HMC had a hollow structure, and its surface contained channels (Fig. 2B). The average size of HMC was 158.74 ± 11.42 nm from 20 nanoparticles. Elemental analysis showed that HMC contained a large amount of C, N, and O (Fig. S3). Further, elemental mapping demonstrated that C, N, and O were largely enriched on the surface of HMC, further demonstrating the hollow structure of these particles (Fig. 2C). X-ray diffraction analyses indicated that HMC did not have any obvious crystalline structure (Fig. 2D), and it was a carbon nanomaterial. Brunauer–Emmett–Teller analysis showed that HMC exhibited good nitrogen adsorption effects (Fig. 2E), with a maximum saturated adsorption capacity of 304.25 cm^3^/g. The adsorption/desorption curve of HMC conformed to a type IV adsorption isotherm, consistent with the H4 type. The hysteresis loop isotherm did not show an obvious saturated adsorption plateau, in line with the irregularity observed in the pore size test (Fig. S4). The average pore diameter is 5.3 ± 0.4 nm, which can be used to delivery small molecular. Overall, the findings showed that ZIF-8 collapsed randomly at high temperatures to generate carbon structures, making forming regular channels remain challenging.

**Fig. 3.**
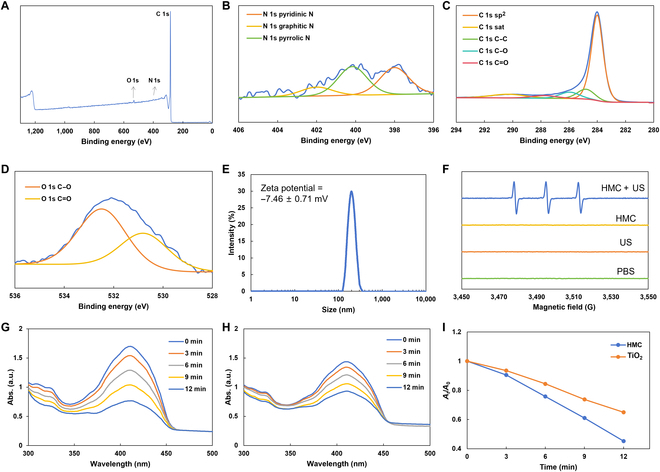
Structural analysis of HMC and the impact of sonodynamic treatment. (A) Full X-ray photoelectron spectroscopy spectrum of HMC. (B) N 1s spectrum of HMC. (C) C 1s spectrum of HMC. (D) O 1s spectrum of HMC. (E) Particle size distribution and zeta potential of HMC in an aqueous suspension. (F) Electron paramagnetic resonance spectra of HMC aqueous suspensions under different conditions. (G) Time-dependent degradation of 1,3-diphenylisobenzofuran (DPBF) by HMC in the presence of ultrasonic treatment. (H) Time-dependent degradation of DPBF by TiO_2_ in the presence of ultrasonic treatment. (I) Normalized results showing the ability of HMC and TiO_2_ to degrade DPBF under prolonged ultrasonication. sat., saturated; Abs., absorbance; PBS, phosphate-buffered saline.

X-ray photoelectron spectroscopy was employed to elucidate the microstructure of HMC (Fig. 3A to D). The results showed that HMC contained C, N, and O, which was consistent with the results of the elemental analysis. The analysis of the N 1s spectra showed that HMC mainly contained 3 types of N elements: pyridinic, graphitic, and pyrrolic. This indicated that HMC was primarily composed of porphyrin-like N structures and graphitic N, suggesting that it may have a sonodynamic effect similar to that of porphyrin. The analysis of the C 1s and O 1s spectra revealed that HMC was mainly composed of sp^2^ C and various functional groups formed by C and O, indicating that HMC had a more typical composition of carbon nanomaterials.

Subsequently, dynamic light scattering analysis and zeta potential tests were performed to examine HMC nanoparticles dispersed in an aqueous medium. HMC could be effectively dispersed in an aqueous medium, with a hydrated particle size of 185.72 ± 17.4 nm and a zeta potential of −7.46 ± 0.71 mV (Fig. 3E). These results also indicated that HMC remained stable in blood. In order to test the sonodynamic effect of HMC, the electron paramagnetic resonance spectra were tested under different conditions. 5,5-Dimethyl-1-pyrroline *N*-oxide was used as a radical scavenger. The results showed that only HMC demonstrated obvious electron paramagnetic resonance peaks under US treatment, and the corresponding peak shapes and ratios indicated that the active substance produced was singlet oxygen (Fig. 3F). The ability of HMC to produce singlet oxygen and degrade 1,3-diphenylisobenzofuran under different durations of ultrasonication was analyzed using commercial TiO_2_ as a control (Fig. 3G to I). HMC could degrade more 1,3-diphenylisobenzofuran within the same time (degradation rate = 54.8% vs. 35.0%) than TiO_2_. This showed that HMC had better singlet-oxygen-generating capacity and could produce more ROS per unit time, thus providing a better tumoricidal effect.

### Phagocytosis and tumor cell death induced by HMC

**Fig. 4.**
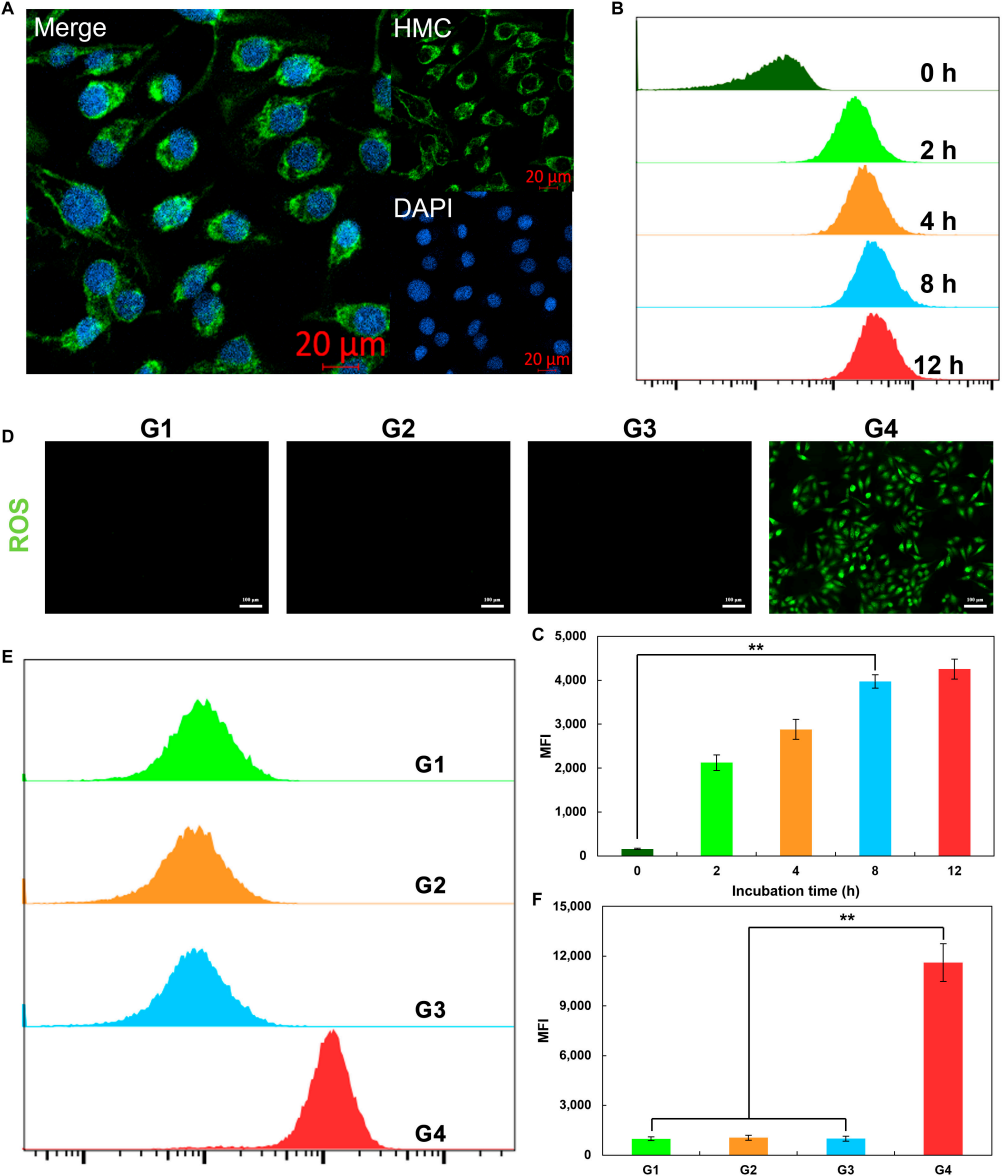
Entry of HMC into tumor cells and its ability to generate ROS in response to ultrasonic treatment. (A) Confocal images of HMC in PAN02 cells after 8 h of co-incubation. Scale bar: 20 μm. (B) Flow cytometric analysis and (C) quantitative analysis of HMC and PAN02 cells after co-incubation for different durations. (D) Fluorescence images of intracellular ROS production by HMC under different conditions. Scale bar: 100 μm. (E) Flow cytometric analysis and (F) quantitative analysis (G1, PBS; G2, US; G3, HMC; G4, HMC + US) (*n* = 3, mean ± SD). DAPI, 4′,6-diamidino-2-phenylindole; MFI, mean fluorescence intensity.

Our experiments showed that HMC could effectively generate singlet oxygen after ultrasonication and thus had tumoricidal potential. However, their tumoricidal effects were dependent on their ability to effectively enter cells, which remained to be tested. First, fluorescein isothiocyanate was used as a fluorescent marker and loaded into HMC nanoparticles to study their intracellular distribution and phagocytosis after co-incubation with cells (Fig. 4A to C). Confocal microscopy revealed that after 8 h of incubation, HMC was widely distributed throughout the cytoplasm, without showing marked accumulation in specific subcellular organelles. This indicated that HMC nanoparticles efficiently entered the cells and primarily localized to the cytoplasm. Further analysis using flow cytometry was performed to examine the intracellular content of HMC at various time points after incubation. The results demonstrated that the phagocytosis of HMC by cells increased over time. However, there was no significant increase between 8 and 12 h of co-incubation. This finding suggested that the peak of phagocytosis occurred at 8 h. Thus, this time point was chosen for subsequent experiments. Additionally, the ability of HMC to generate ROS in tumor cells was also evaluated (Fig. 4D to F). HMC nanoparticles effectively produced ROS in response to ultrasonic treatment after 8 h of co-incubation with cells. The rate of ROS production in these cells was substantially different from that in control cells. Flow cytometry showed that HMC combined with US treatment (HMC + US) increased intracellular ROS levels by 15-fold. This increase in ROS production was expected to effectively kill and inhibit the growth of tumor cells.

**Fig. 5.**
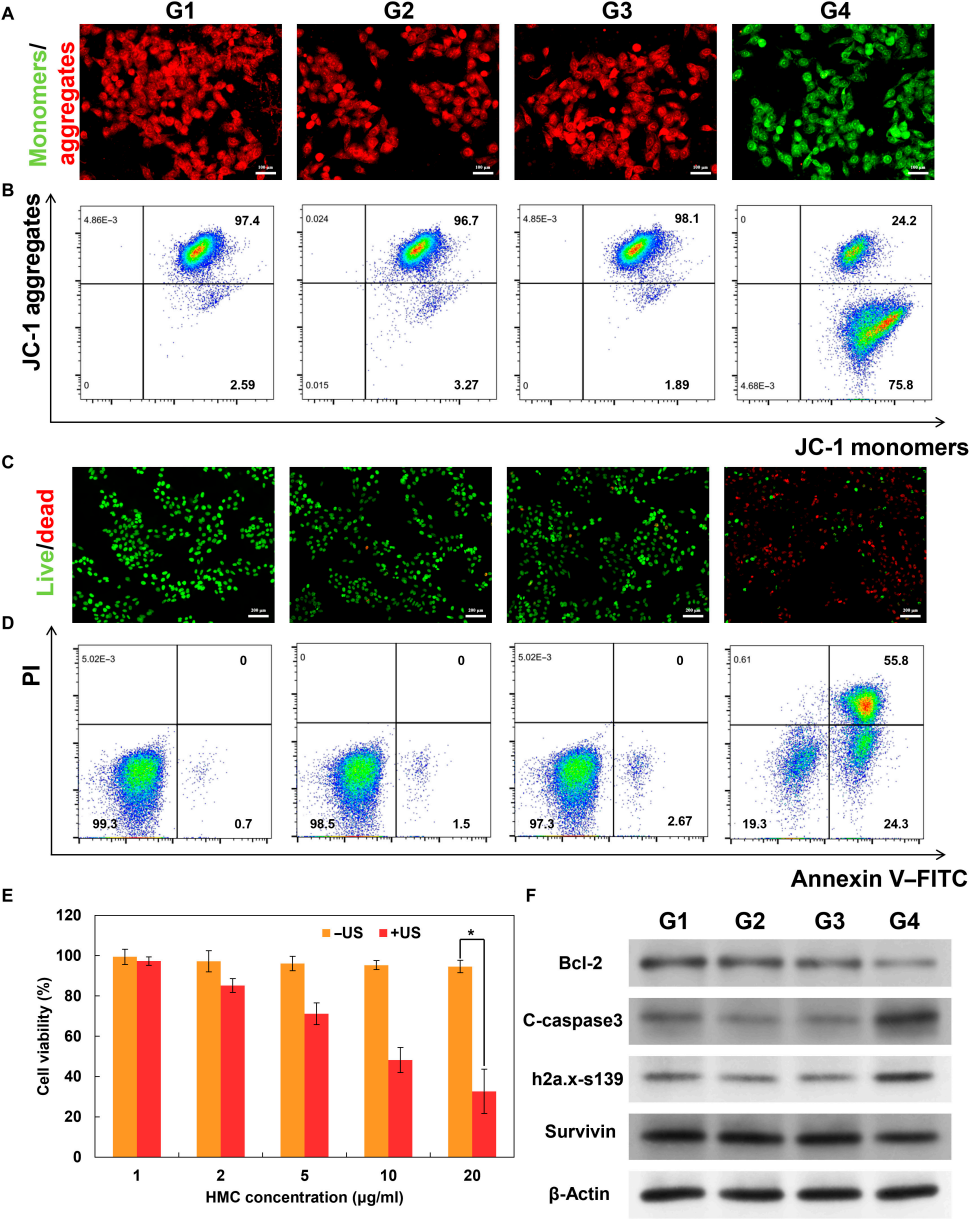
Sonodynamic killing effect of HMC on PAN02 cells and the corresponding mechanism of action. (A) Fluorescence images of JC-1-labeled PAN02 cells after different treatments. Scale bar: 100 μm. (B) Flow cytometry assessments of PAN02 cells stained with JC-1 after different treatments. (C) Representative images of calcein-AM/propidium iodide (PI)-stained PAN02 cells after different treatments. Scale bar: 100 μm. (D) Apoptosis of PAN02 cells after different treatments. (E) Effect of US on cell survival rates after the co-incubation of different concentrations of HMC with PAN02 cells for 8 h. (F) Western blot results showing the expression of apoptosis-related proteins in PAN02 cells after different treatments (G1, PBS; G2, US; G3, HMC; G4, HMC + US) (*n* = 3, mean ± SD).

The impact of HMC + US on mitochondrial membrane potential was also investigated. A marked decrease in mitochondrial membrane potential was detected when the cells were treated with both HMC and US. Flow cytometry results indicated that approximately 75.8% of the JC-1 probes existed as monomers after treatment, in contrast to <4% in other groups (Fig. 5A and B and Fig. S5). This suggested that the singlet oxygen generated by HMC in response to US treatment notably reduced the mitochondrial membrane potential, impairing the ability of mitochondria to maintain normal physiological functions in tumor cells and potentially inducing early apoptosis. Live/dead fluorescence staining confirmed that HMC + US treatment effectively killed tumor cells (Fig. 5C and Fig. S6). Further analysis showed that 24.3% of the cells in the HMC + US group were in the early apoptosis phase, while 55.8% were in the late apoptosis or necrosis phase (Fig. 5D). These results were consistent with the observed decrease in mitochondrial membrane potential. Additionally, cell viability was assessed using the Cell Counting Kit-8 assay before and after US treatment (Fig. 5E). The cell survival rate was lower than 40% after treatment with 20 μg/ml HMC combined with US, which was consistent with the abovementioned results. Western blot analysis also revealed that in the HMC + US group, the expression of the apoptosis-inhibiting proteins Bcl-2 (from 0.345 to 0.154) and survivin (from 1.268 to 0.947) was down-regulated, while that of the apoptosis-promoting proteins C-caspase3 (from 0.152 to 0.249) and γH2AX-S139 (from 0.248 to 0.381) was up-regulated (Fig. 5F and Table S1). These findings further demonstrated that HMC + US treatment induces tumor cell death by promoting apoptosis.

### HMC promotes the maturation of DCs in vitro

**Fig. 6.**
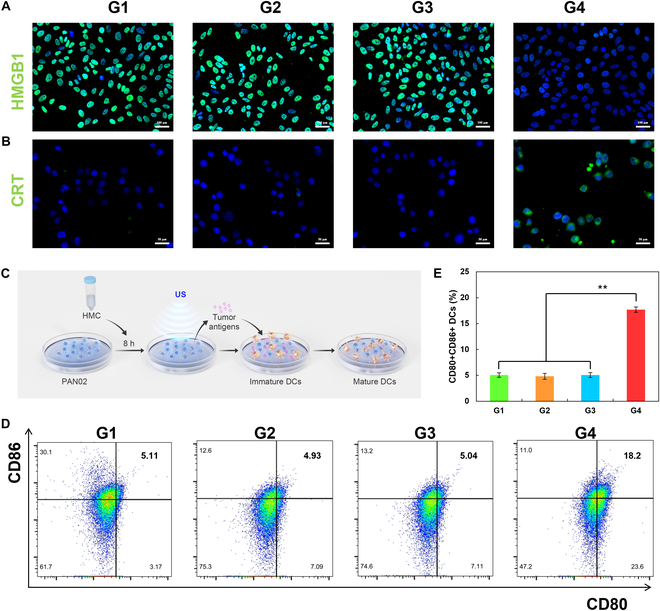
Release of immune factors and the activation of DCs in vitro after the treatment of PAN02 cells with HMC. Fluorescence microscopy of the cytokines (A) HMGB1 (scale bar: 100 μm) and (B) CRT (scale bar: 50 μm) in PAN02 cells after different treatments. (C) Schematic diagram of the experiment conducted to examine the activation of DCs after different treatments. (D) Flow cytometric analysis and (E) quantitative analysis of DC activation under different treatment conditions (G1, PBS; G2, US; G3, HMC; G4, HMC + US) (*n* = 3, mean ± SD).

Our previous experiments showed that HMC can induce apoptosis under US treatment and thereby produce tumoricidal effects. During apoptosis, tumor cells secrete cytokines that promote the maturation of DCs and thus enhance autoimmune responses, thereby achieving tumor immunotherapy. Hence, the expressions of CRT, high mobility group protein B1 (HMGB1), and released adenosine triphosphate (ATP) in PAN02 cells were examined under different treatment conditions (Fig. 6A and B and Fig. S7). Confocal imaging and immunofluorescence staining showed that after HMC + US treatment, HMGB1 was down-regulated and CRT was up-regulated in PAN02 cells. Further, the released ATP was increased from 0.39 to 1.63 nM under different treatment conditions. This suggested that HMGB1, CRT, and ATP were released from tumor cells and entered the culture medium in the upper chamber. Thereafter, the ability of the upper chamber culture medium to promote DC maturation in vitro was tested (Fig. 6C). Flow cytometry demonstrated that the rate of DC maturation increased from 5.11% to 18.2% after co-cultivation with the upper chamber culture medium (Fig. 6D and E). This suggested that after HMC + US treatment, apoptotic tumor cells could promote the maturation of DCs by releasing HMGB1 and CRT, potentially enhancing the effects of immunotherapy.

### HMC enhanced sono-immunotherapy effects in vivo

**Fig. 7.**
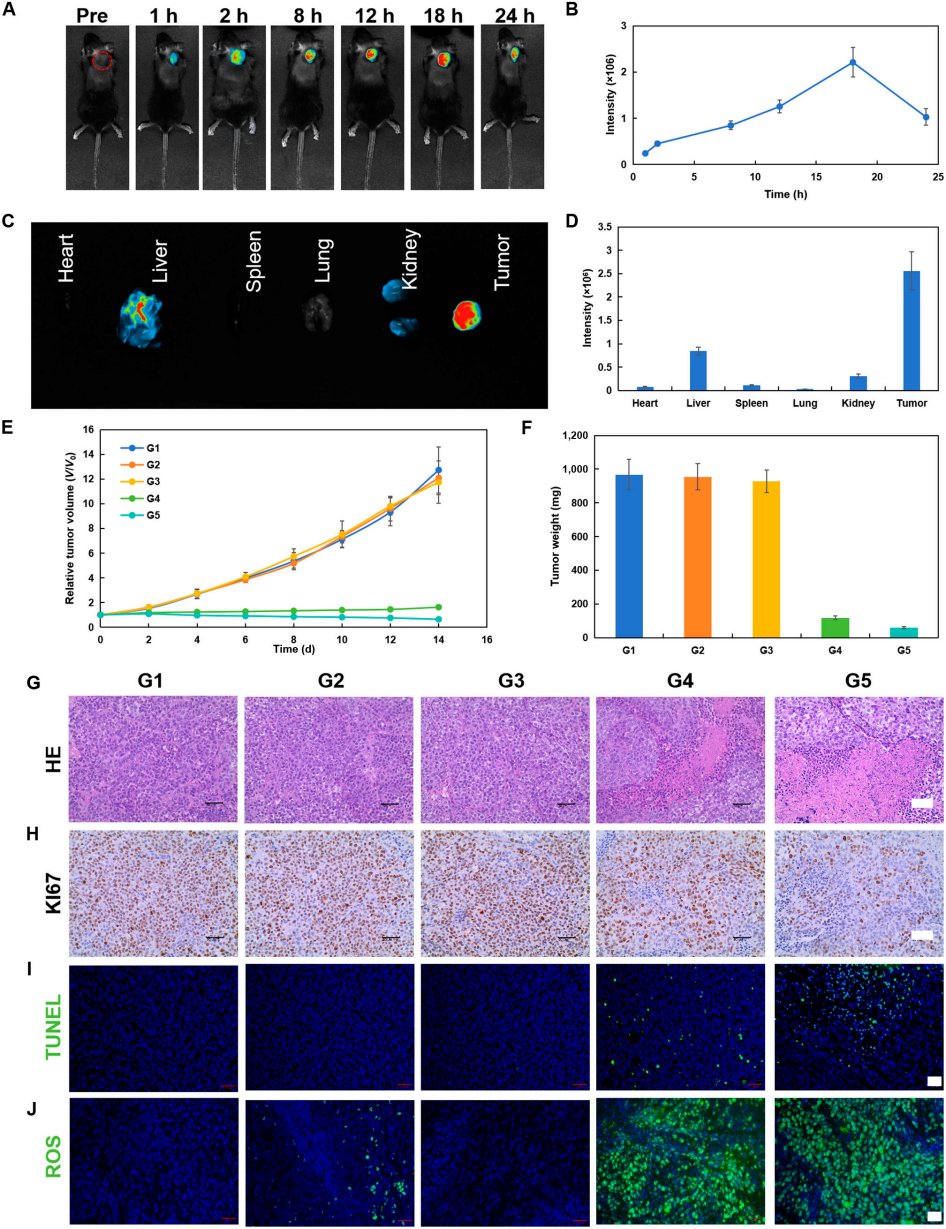
In vivo distribution of HMC and evaluation of in vivo tumor inhibition. (A) Time-dependent in vivo distribution of HMC in PAN02 tumor-bearing mice following the injection of HMC via the tail vein. (B) Time-dependent fluorescence intensity curves generated after the tail vein injection of HMC. (C) Fluorescence imaging and (D) fluorescence intensity curves generated from main organs at 18 h after the tail vein injection of HMC. (E) Changes in tumor volume in mice receiving different treatments for 14 d. (F) Tumor masses in mice receiving different treatments for 14 d. (G) Hematoxylin and eosin (H&E) (scale bar: 50 μm), (H) KI67 (scale bar: 50 μm), and (I) terminal deoxynucleotidyl transferase dUTP nick end labeling (TUNEL) (scale bar: 20 μm) staining of pancreatic tumor tissues from mice undergoing different treatments for 12 d. (J) ROS levels in tumors obtained from mice undergoing different treatments. Scale bar: 20 μm (G1, PBS; G2, US; G3, PD-L1-IN-1@HMC; G4, HMC + US; G5, PD-L1-IN-1@HMC + US) (*n* = 5, mean ± SD).

The biosafety of HMC was investigated. Specifically, mice were treated thrice with either phosphate-buffered saline (PBS) or HMC (50 mg/kg) via tail vein injections. None of the mice died over a 14-d period posttreatment. Subsequently, the main organs (e.g., heart, liver, spleen, lungs, kidneys, and brain) of the mice were removed, sectioned, and analyzed (Fig. S8). Comparisons with the PBS group showed that the injection of HMC did not cause any irreversible damage to the main organs, suggesting that HMC had good biosafety and could be used to inhibit tumor growth in vivo. In order to determine the optimal timing of ultrasonic irradiation, the distribution of HMC in vivo was tested via the tail vein injection of cyanine5-labeled HMC (Fig. 7A to D). The enrichment of HMC in tumors was found to peak at 18 h after injection, suggesting that 18 h after injection was the optimal time point for ultrasonic irradiation. Notably, small-molecule inhibitors of PD-L1 (PD-L1-IN-1) were loaded into HMC to enhance the effects of immunotherapy. The feasibility of sono-immunotherapy was subsequently tested in PAN02 tumor-bearing mice after different treatments (G1, PBS; G2, US; G3, PD-L1-IN-1@HMC; G4, HMC + US; G5, PD-L1-IN-1@HMC + US). In the US groups, ultrasonic irradiation was performed at 18 h after injection. No obvious decrease in body weight was observed in any group during the treatment period, demonstrating that the treatment process did not cause any significant physiological impairments (Fig. S8). Fig. 7E and F show that there was no difference in tumor volume among G1, G2, and G3 mice. However, tumor growth was slower in G4 and G5 mice, and the tumor size in G5 mice was markedly reduced due to the combined effect of PD-L1-IN-1. Overall, HMC could effectively inhibit tumor growth following US treatment, and PD-L1-IN-1 could enhance this inhibitory effect even further. Hence, sono-immunotherapy was effectively achieved in G5 mice, resulting in the effective inhibition of tumor growth through the combination of HMC and PD-L1-IN-1. Subsequently, tumor tissues from different groups of mice were sectioned and analyzed. Hematoxylin and eosin staining revealed the presence of a greater number and density of tumor cells in G1, G2, and G3, while G4 and G5 contained large regions of tumor necrosis. The region of tumor necrosis was higher in G5 than in G4 (Fig. 7G). KI67 and terminal deoxynucleotidyl transferase dUTP nick end labeling (TUNEL) staining also confirmed the strong tumoricidal and inhibitory effects in G4 and G5, with the therapeutic effect in G5 being better than that in G4 (Fig. 7H and I). Finally, ROS fluorescence analysis also revealed that large amounts of ROS were generated following US treatment in G4 and G5, and the ROS generated could effectively kill tumor cells and inhibit tumor growth (Fig. 7J).

**Fig. 8.**
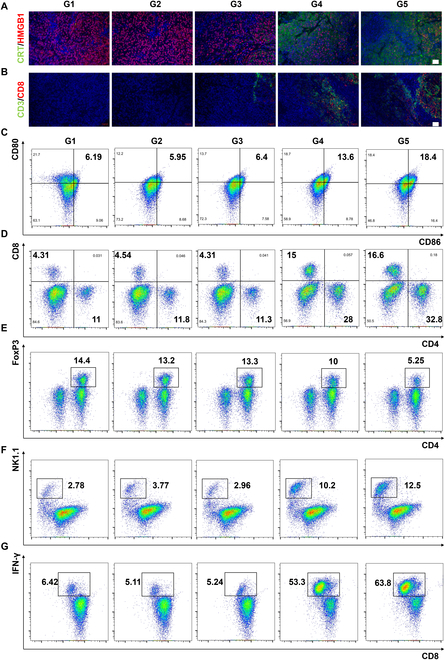
Immune responses induced by HMC treatment in vivo. (A and B) Fluorescence images showing (A) CRT and HMGB1 expression and (B) CD3/CD8 expression at the tumor site in PAN02 tumor-bearing mice after different treatments, Scale bar: 20 μm. (C) Proportion of mature DCs (CD45+ CD11b+ MHCII+ CD80+ CD86+) in the draining lymph nodes of tumor-bearing mice detected via flow cytometry. (D) Proportion of CD8+ T cells (CD45+ CD3+ CD8+) and CD4+ T cells (CD45+ CD3+ CD4+) in tumor tissues. (E) Proportion of regulatory T (Treg) cells (CD45+ CD3+ CD4+ FoxP3+) in tumor tissues. (F) Proportion of natural killer (NK) cells (CD45+ CD3-NK1.1+) in tumor tissues. (G) Proportion of activated CD8+ T cells (CD45+ CD3+ CD8+ IFN-γ+) in tumor tissues (G1, PBS; G2, US; G3, PD-L1-IN-1@HMC; G4, HMC + US; G5, PD-L1-IN-1@HMC + US). IFN-γ, interferon gamma.

In order to investigate the effects of immunotherapy, the expression of CRT and HMGB1 in tumor cells was analyzed across different groups of mice (Fig. 8A and Fig. S9). The amount of CRT increased after treatment, while that of HMGB1 decreased considerably after HMC and US treatment, consistent with the results of in vitro tests. The findings demonstrated that both the G4 and G5 treatments promoted the release of immune factors, thereby enhancing the efficacy of immunotherapy. Fluorescence immunoassays for CD3 and CD8 revealed that expression of CD3 and CD8 in tumors increased following sonodynamic therapy, potentially inducing immunological effects that could inhibit tumor growth (Fig. 8B and Fig. S10). Thus, the content of different cell types in the lymph nodes and tumors of mice were quantified (Fig. 8C to G). First, the mature DCs in the lymphatic vessels of mice were enumerated. The content of mature DCs was much higher in G4 and G5 than in the other 3 groups, and PD-L1-IN-1 enhanced the number of DCs, thus promoting immunological responses. Subsequently, the number of CD4+ and CD8+ T cells in tumors was analyzed. Sonodynamic therapy was found to effectively increase the number of CD4+ and CD8+ T cells in tumors, and PD-L1-IN-1 further enhanced this effect. Notably, the number of regulatory T cells in tumors showed a decrease (from 14.4% to 5.25%) in G4 and G5, suggesting that sonodynamic therapy may also increase the efficacy of immunotherapy by disrupting the immunosuppressive tumor microenvironment. The number of natural killer cells and interferon gamma-positive CD8+ T cells also increased after sonodynamic therapy combined with PD-L1-IN-1 treatment, further reflecting the presence of more active immune cells in tumors. This could lead to enhanced tumoricidal effects and improve the effectiveness of immunotherapy. Overall, the therapeutic effect in G4 mice was derived from sonodynamic treatment alone, which directly killed tumor cells, activated the immune system, and disrupted the state of immunosuppression in the tumor microenvironment. However, after incorporating a small-molecule inhibitor of PD-L1, the effects of immunotherapy could be enhanced, thus realizing sono-immunotherapy. This treatment was even more effective at eliminating tumor cells and inhibiting tumor tissues.

**Fig. 9.**
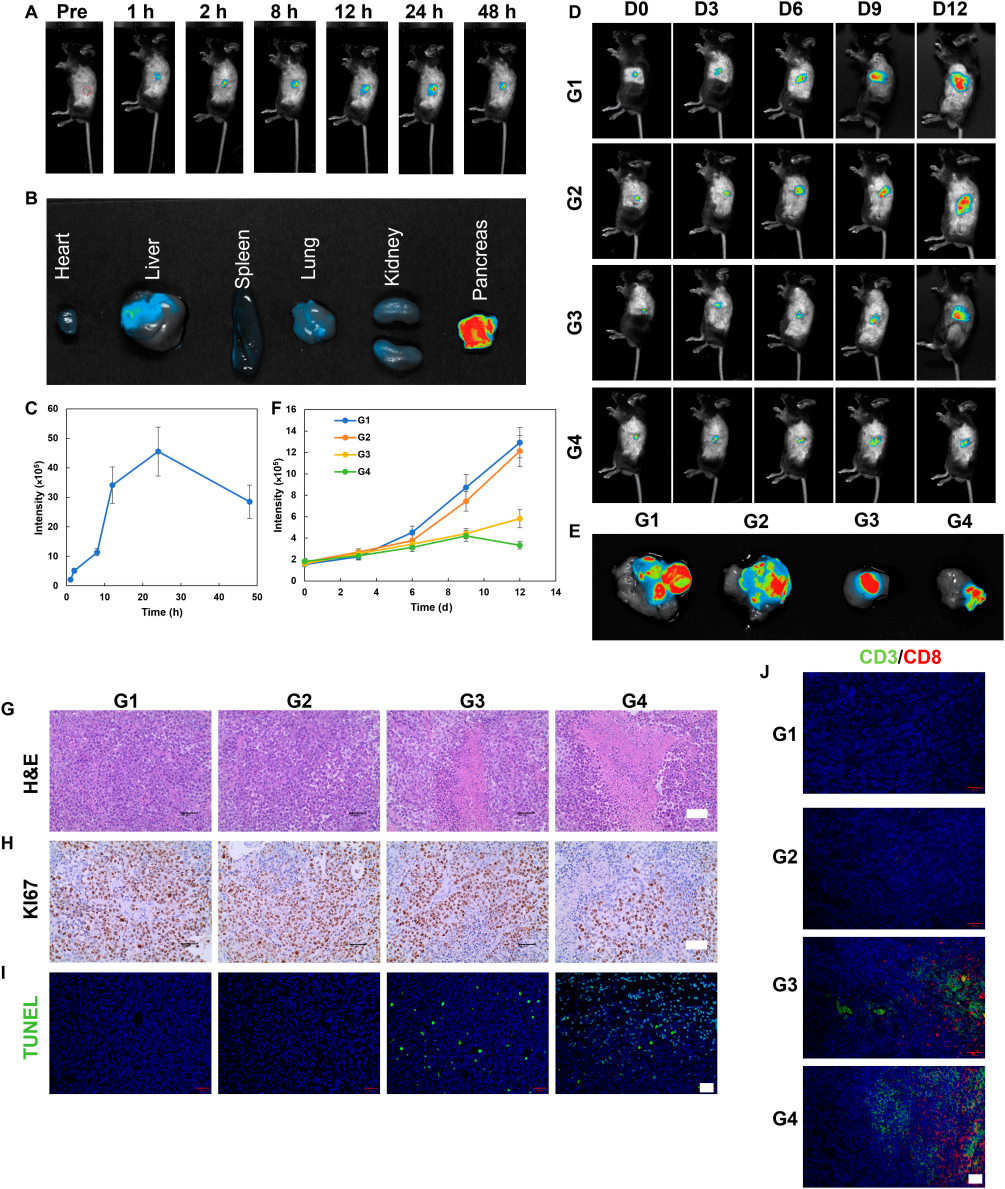
Treatment of in situ pancreatic cancer tumors with HMC. (A) Time-dependent in vivo distribution of HMC in mice with in situ pancreatic tumors following the injection of HMC via the tail vein. (B) Fluorescence imaging of various organs after the tail vein injection of HMC. (C) Time-dependent fluorescence intensity curves generated after the tail vein injection of HMC. (D) In vivo fluorescence intensity in mice after different treatments (fluorescence from PAN02-LUCI). (E) Ex vivo fluorescence analysis of the pancreas on day 12 after different treatments. (F) Quantitative analysis of in vivo fluorescence intensity in mice at various time points after different treatments. (G) H&E (scale bar: 50 μm), (H) KI67 (scale bar: 50 μm), (I) TUNEL (scale bar: 20 μm), and (J) CD3/CD8 (scale bar: 20 μm) immunofluorescence staining of pancreatic tumor tissues from mice after 12 d of different treatments (G1, PBS; G2, US; G3, HMC + US; G4, PD-L1-IN-1@HMC + US) (*n* = 5, mean ± SD).

To determine the in vivo therapeutic effect of HMC, an in situ pancreatic cancer model was constructed. To determine the optimal timing of ultrasonication, the in vivo distribution of cyanine5-labeled HMC was tested after tail vein injection (Fig. 9A to C). The highest enrichment of HMC in the in situ pancreatic cancer model was observed at 24 h after injection. Ex vivo tests showed that the injected HMC nanoparticles were enriched in the pancreas, which was conducive to sonodynamic treatment. The efficacy of sono-immunotherapy was evaluated in mice with in situ pancreatic cancer (Fig. 9D to F). The mice were divided into the following groups and received treatment injections on days 0, 2, and 4—G1, PBS; G2, US; G3, HMC + US; and G4, PD-L1-IN-1@HMC + US. In the US groups, ultrasonic irradiation was performed 24 h after injection. The mice were observed over an experimental duration of 12 d. As treatment progressed, the fluorescence intensity in the pancreas changed. A rapid increase in fluorescence intensity was observed in the G1 and G2 groups, while the G3 and G4 groups showed a slower increase in fluorescence intensity. In other words, the G3 and G4 treatments could effectively inhibit the growth of pancreatic tumors when compared with the G1 and G2 treatments. Hence, sonodynamic therapy could effectively inhibit the growth of in situ pancreatic cancer. Notably, the fluorescence intensity of pancreatic tumors was similar between G3 and G4 during the first 9 d of treatment, but the fluorescence intensity in G4 became lower than that in G3 toward the end of the treatment period. This suggested that the PD-L1 inhibitor could enhance the tumoricidal activity of T cells by inhibiting the expression of PD-L1 in tumor cells. This not only enhanced the effects of immunotherapy but also enabled the long-term elimination of tumor cells and the inhibition of in situ pancreatic cancer. Overall, sono-immunotherapy offered good therapeutic effects against both back tumors and in situ pancreatic cancer.

Additionally, pancreatic tumor sections from different groups were analyzed (Fig. 9G to J). On hematoxylin and eosin staining, G1 and G2 showed a greater number and density of tumor cells, while G3 and G4 showed a large area of necrotic tissue. In fact, G4 had a larger area of tumor necrosis than G3. KI67 and TUNEL staining also demonstrated the superior tumoricidal and tumor inhibition capacity of the G3 treatments, with G4 having a better therapeutic effect than G3. Combined with the observed expression of CD3 and CD8 in tumors, the results showed that the release of PD-L1 small-molecule inhibitors in response to US could improve the efficacy of sono-immunotherapy and prolong the duration of action, resulting in a better therapeutic effect against in situ pancreatic cancer.

Overall, HMC appeared to be suitable for injection owing to its good biocompatibility, and it could accumulate in both back tumors and in situ pancreatic cancer tissues. Subsequently, it could generate ROS to kill tumor cells following US treatment and effectively release PD-L1 small-molecule inhibitors to enhance the effects of immunotherapy via sonodynamic treatment. Animal experiments revealed that HMC nanoparticles could effectively eliminate malignant cells and inhibit the growth of pancreatic cancer after US treatment. Furthermore, owing to their hollow and porous structure, they could carry not only small-molecule inhibitors but also other small compounds based on the specific treatment needs in various clinical scenarios. Hence, these nanoparticles could enhance the elimination of malignant tumors in conjunction with other methods while exerting their own sonodynamic treatment effects.

## Conclusion

To enhance the efficacy of sono-immunotherapy for pancreatic cancer, HMC nanoparticles with a hollow and mesoporous structure were developed in this study using a calcination-etching method with SiO_2_@ZIF-8 as the sacrificial template. These HMC nanoparticles had a porphyrin-like structure and demonstrated a potential for sonodynamic treatment. Degradation experiments showed that HMC produced singlet oxygen more effectively than commercial TiO_2_. In vitro tests revealed that HMC nanoparticles not only entered tumor cells but also generated ROS following US exposure. This ROS production led to apoptosis and the death of tumor cells, effectively inhibiting tumor cell growth. Additionally, HMC stimulated tumor cells to secrete immune factors and promoted DC maturation, contributing to immunological effects. In vivo tests demonstrated that HMC nanoparticles, when combined with a PD-L1 inhibitor, could substantially inhibit tumor growth and activate immune responses in mice following US treatment, enhancing the effectiveness of sono-immunotherapy. The in situ pancreatic cancer model further confirmed that HMC nanoparticles loaded with a PD-L1 inhibitor could effectively suppress tumor growth following US treatment. Overall, this study introduces a novel method for the fabrication of HMC nanoparticles with sonodynamic properties using a sacrificial template method. The findings highlight the potential clinical applications of these nanoparticles in tumor treatment. Additionally, the unique structure of the HMC allows the loading of different molecules. Thus, they could be combined with various therapies for tumor growth inhibition.

## Ethical Approval

All animal experiments were carried out after approval by the ethical committee for animal care of Ningbo University (Permit No. SYXK ZHE 2022-0028).

## Data Availability

Data are provided within the article or Supplementary Materials files.

## References

[1] Bray F, Laversanne M, Sung H, Ferlay J, Siegel RL, Soerjomataram I, Jemal A. Global cancer statistics 2022: GLOBOCAN estimates of incidence and mortality worldwide for 36 cancers in 185 countries. CA-Cancer J Clin. 2022;74(2024):229–263. 10.3322/caac.21834 38572751

[2] Islami F, Marlow EC, Thomson B, McCullough ML, Rumgay H, Gapstur SM, Patel AV, Soerjomataram I, Jemal A. Proportion and number of cancer cases and deaths attributable to potentially modifiable risk factors in the United States, 2019. CA-Cancer J Clin. 2024;74(5):405–432. 38990124 10.3322/caac.21858

[3] Peery AF, Crockett SD, Murphy CC, Lund JL, Dellon ES, Williams JL, Jensen ET, Shaheen NJ, Barritt AS, Lieber SR, et al. Burden and cost of gastrointestinal, liver, and pancreatic diseases in the United States: Update 2018. Gastroenterology. 2019;156(1):254–272.e11. 30315778 10.1053/j.gastro.2018.08.063 PMC6689327

[4] Mizrahi JD, Surana R, Valle JW, Shroff RT. Pancreatic cancer. Lancet. 2020;395:2008–2020. 32593337 10.1016/S0140-6736(20)30974-0

[5] Daly MB, Pal T, Berry MP, Buys SS, Dickson P, Domchek SM, Elkhanany A, Friedman S, Goggins M, Hutton ML, et al. Genetic/Familial High-Risk Assessment: Breast, Ovarian, and Pancreatic, version 2.2021, NCCN Clinical Practice Guidelines in Oncology. J Natl Compr Canc Netw. 2021;19(1):77–102. 33406487 10.6004/jnccn.2021.0001

[6] Li L, Li X, Ouyang B, Mo H, Ren H, Yang S. Three-dimensional collision avoidance method for robot-assisted minimally invasive surgery. Cyborg Bionic Syst. 2023;4: Article 0042. 37675200 10.34133/cbsystems.0042 PMC10479965

[7] Sahai E, Astsaturov I, Cukierman E, DeNardo DG, Egeblad M, Evans RM, Fearon D, Greten FR, Hingorani SR, Hunter T, et al. A framework for advancing our understanding of cancer-associated fibroblasts. Nat Rev Cancer. 2020;20:174–186. 31980749 10.1038/s41568-019-0238-1 PMC7046529

[8] Bejarano L, Jordao MJC, Joyce JA. Therapeutic targeting of the tumor microenvironment. Cancer Discov. 2021;11(4):933–959. 33811125 10.1158/2159-8290.CD-20-1808

[9] Biffi G, Tuveson DA. Diversity and biology of cancer-associated fibroblasts. Physiol Rev. 2021;101(1):147–176. 32466724 10.1152/physrev.00048.2019 PMC7864232

[10] Riquelme E, Zhang Y, Zhang LL, Montiel M, Zoltan M, Dong WL, Quesada P, Sahin I, Chandra V, San Lucas A, et al. Tumor microbiome diversity and composition influence pancreatic cancer outcomes. Cell. 2019;178(4):795–806.e12. 31398337 10.1016/j.cell.2019.07.008 PMC7288240

[11] Mao XQ, Xu J, Wang W, Liang C, Hua J, Liu J, Zhang B, Meng QC, Yu XJ, Shi S. Crosstalk between cancer-associated fibroblasts and immune cells in the tumor microenvironment: New findings and future perspectives. Mol Cancer. 2021;20(1):31–24. 34635121 10.1186/s12943-021-01428-1 PMC8504100

[12] Ho WJ, Jaffee EM, Zheng L. The tumour microenvironment in pancreatic cancer—Clinical challenges and opportunities. Nat Rev Clin Oncol. 2020;17(9):527–540. 32398706 10.1038/s41571-020-0363-5 PMC7442729

[13] Chen YB, Song YC, Du W, Gong LL, Chang HC, Zou ZZ. Tumor-associated macrophages: An accomplice in solid tumor progression. J Biomed Sci. 2019;26(1): Article 78. 10.1186/s12929-019-0568-z PMC6800990 31629410

[14] Liu TY, Han CC, Wang SW, Fang PQ, Ma ZF, Xu L, Yin R. Cancer-associated fibroblasts: An emerging target of anti-cancer immunotherapy. J Hematol Oncol. 2019;12(1): Article 86. 31462327 10.1186/s13045-019-0770-1 PMC6714445

[15] Li J, Luo Y, Zeng Z, Cui D, Huang J, Xu C, Li L, Pu K, Zhang R. Precision cancer sono-immunotherapy using deep-tissue activatable semiconducting polymer immunomodulatory nanoparticles. Nat Commun. 2022;13(1): Article 4032. 35821238 10.1038/s41467-022-31551-6 PMC9276830

[16] Chen SY, Ding JH, Bekeredjian R, Yang BZ, Shohet RV, Johnston SA, Hohmeier HE, Newgard CB, Grayburn PA. Efficient gene delivery to pancreatic islets with ultrasonic microbubble destruction technology. Proc Natl Acad Sci USA. 2006;103(22):8469–8474. 16709667 10.1073/pnas.0602921103 PMC1482516

[17] Rapoport N, Nam KH, Christensen DA, Kennedy AM, Shea JE, Scaife CL. Ultrasound-enhanced nanotherapy of pancreatic cancer. AIP Conf Proc. 2009;1215:123–126.

[18] Sofuni A, Asai Y, Mukai S, Yamamoto K, Itoi T. High-intensity focused ultrasound therapy for pancreatic cancer. J Med Ultrason. 2022; 10.1007/s10396-022-01208-4. 10.1007/s10396-022-01208-4 35551555

[19] Ilbeigi S, Ranjbar A, Zahraie N, Vais RD, Monjezi MR, Sattarahmady N. Sonodynamic therapy of pancreatic cancer cells based on synergistic chemotherapeutic effects of selenium-PEG-curcumin nanoparticles and gemcitabine. Appl Phys A Mater Sci Process. 2023;129: Article 82.

[20] Zhang T, Sun Y, Cao J, Luo J, Wang J, Jiang Z, Huang P. Intrinsic nucleus-targeted ultra-small metal–organic framework for the type I sonodynamic treatment of orthotopic pancreatic carcinoma. J Nanobiotechnology. 2021;19(1): Article 315. 34641905 10.1186/s12951-021-01060-7 PMC8507249

[21] Liang S, Yao JJ, Liu D, Rao L, Chen XY, Wang ZH. Harnessing nanomaterials for cancer sonodynamic immunotherapy. Adv Mater. 2023;35(33): Article 2211130. 10.1002/adma.202211130 36881527

[22] Zhang C, Pu KY. Organic sonodynamic materials for combination cancer immunotherapy. Adv Mater. 2023;35(51): Article e2303059. 37263297 10.1002/adma.202303059

[23] Ding M, Zhu A, Zhang Y, Liu J, Lin L, Wang X, Li J. Neutrophil-based Trojan horse containing polymer nano-therapeutics for sono-activatable ferroptosis-immunotherapy of orthotopic glioma. Nano Today. 2024;57: Article 102398.

[24] Yu N, Zhou J, Ding M, Li M, Peng S, Li J. Sono-triggered cascade lactate depletion by semiconducting polymer nanoreactors for cuproptosis-immunotherapy of pancreatic cancer. Angew Chem Int Ed Engl. 2024;63(30): Article e202405639. 38708791 10.1002/anie.202405639

[25] Yang Y, Cheng Y, Cheng L. The emergence of cancer sono-immunotherapy. Trends Immunol. 2024;45(7):549–563. 38910097 10.1016/j.it.2024.06.001

[26] Zhao C, Tang X, Zhao J, Cao J, Jiang Z, Qin J. MOF derived core-shell CuO/C with temperature-controlled oxygen-vacancy for real time analysis of glucose. J Nanobiotechnology. 2022;20(1): Article 507. 36456946 10.1186/s12951-022-01715-z PMC9714170

[27] Yang WP, Li XX, Li Y, Zhu RM, Pang H. Applications of metal-organic-framework-derived carbon materials. Adv Mater. 2019;31(6): Article 1804740. 10.1002/adma.201804740 30548705

[28] Zhang Y, Lv QY, Chi K, Li QL, Fan HL, Cai B, Xiao F, Wang S, Wang Z, Wang L. Hierarchical porous carbon heterojunction flake arrays derived from metal organic frameworks and ionic liquid for H_2_O_2_ electrochemical detection in cancer tissue. Nano Res. 2021;14:1335–1343.

[29] Wu YQ, Qiu XC, Liang F, Zhang QK, Koo A, Dai YN, Lei Y, Sun XL. A metal-organic framework-derived bifunctional catalyst for hybrid sodium-air batteries. Appl Catal B. 2019;241:407–414.

[30] Han X, Zhao C, Wang S, Pan Z, Jiang Z, Tang X. Multifunctional TiO_2_/C nanosheets derived from 3D metal–organic frameworks for mild-temperature-photothermal-sonodynamic-chemodynamic therapy under photoacoustic image guidance. J Colloid Interface Sci. 2022;621:360–373. 35462177 10.1016/j.jcis.2022.04.077

[31] Han X, Li Y, Zhou Y, Song Z, Deng Y, Qin J, Jiang Z. Metal-organic frameworks-derived bimetallic nanozyme platform enhances cytotoxic effect of photodynamic therapy in hypoxic cancer cells. Mater Des. 2021;204: Article 109646.

[32] Xiang Z, Qi YY, Lu YS, Hu ZR, Wang X, Jia WW, Hu JZ, Ji JS, Lu W. MOF-derived novel porous Fe_3_O_4_@C nanocomposites as smart nanomedical platforms for combined cancer therapy: Magnetic-triggered synergistic hyperthermia and chemotherapy. J Mater Chem B. 2020;8:8671–8683. 32856668 10.1039/d0tb01021a

[33] Pachfule P, Shinde D, Majumder M, Xu Q. Fabrication of carbon nanorods and graphene nanoribbons from a metal–organic framework. Nat Chem. 2016;8:718–724. 27325100 10.1038/nchem.2515

[34] Geng P, Yu N, Macharia DK, Meng RR, Qiu P, Tao C, Li MQ, Zhang HJ, Chen ZG, Lian WS. MOF-derived CuS@Cu-MOF nanocomposites for synergistic photothermal-chemodynamic-chemo therapy. Chem Eng J. 2022;441: Article 135964.

[35] Zhu S, Xu L, Yang S, Zhou X, Chen X, Dong B, Bai X, Lu G, Song H. Cobalt-doped ZnO nanoparticles derived from zeolite imidazole frameworks: Synthesis, characterization, and application for the detection of an exhaled diabetes biomarker. J Colloid Interface Sci. 2020;569:358–365. 32126348 10.1016/j.jcis.2020.02.081

[36] Zhao C, Kang J, Li Y, Wang Y, Tang X, Jiang Z. Carbon-based stimuli-responsive nanomaterials: Classification and application. Cyborg Bionic Syst. 2023;4: Article 0022. 37223546 10.34133/cbsystems.0022 PMC10202192

[37] Wang DD, Jana DL, Zhao YL. Metal–organic framework derived nanozymes in biomedicine. Acc Chem Res. 2020;53(7):1389–1400. 32597637 10.1021/acs.accounts.0c00268

[38] Chen Z, Du Q, Guo W, Huang H, Li H, Zheng Y, Tan L, Fu C, Wu Q, Ren X, et al. Nanozymes-engineered metal–organic frameworks for enhanced microwave thermodynamic therapy in PDX of hepatic carcinoma. Chem Eng J. 2022;450(Part 2): Article 138092.

[39] Pan XT, Bai LX, Wang H, Wu QY, Wang HY, Liu S, Xu BL, Shi XH, Liu HY. Metal–organic-framework-derived carbon nanostructure augmented sonodynamic cancer therapy. Adv Mater. 2018;30(23): Article 1800180. 10.1002/adma.201800180 29672956

[40] Jiang Z, Han X, Du Y, Li Y, Li Y, Li J, Tian J, Wu A. Mixed metal metal–organic frameworks derived carbon supporting ZnFe_2_O_4_/C for high-performance magnetic particle imaging. Nano Lett. 2021;21(7):2730–2737. 33797257 10.1021/acs.nanolett.0c04455

[41] Wang DD, Wu HH, Lim WQ, Phua SZF, Xu PP, Chen QW, Guo Z, Zhao YL. A mesoporous nanoenzyme derived from metal–organic frameworks with endogenous oxygen generation to alleviate tumor hypoxia for significantly enhanced photodynamic therapy. Adv Mater. 2019;31(27): Article e1901893. 31095804 10.1002/adma.201901893

[42] Huang X, Zhang ST, Tang YJ, Zhang XY, Bai Y, Pang H. Advances in metal–organic framework-based nanozymes and their applications. Coord Chem Rev. 2021;449: Article 214216.

