# Supplementary material for: Hollow Mesoporous Carbon Nanospheres Derived from Metal–Organic Frameworks for Efficient Sono-immunotherapy against Pancreatic Cancer
Source: Cyborg Bionic Syst. 2025 May 9;6:0247. doi: 10.34133/cbsystems.0247 (PMC12062583; doi:10.34133/cbsystems.0247)
Supplement: Supplementary 1 — Supplementary Methods Figs. S1 to S13 Table S1 [file cbsystems.0247.f1.docx]

**Hollow Mesoporous Carbon Nanospheres Derived from Metal–Organic Frameworks for Efficient Sono-Immunotherapy Against Pancreatic Cancer**

Libin Chen^1,2, #^, Haiwei Li^1, #, *^, Jing Liu^3^, Yunzhong Wang^2^, Shengmin Zhang^2^

1.Liaoning Cancer Hospital & Institute, Cancer Hospital of Dalian University of Technology, Cancer Hospital of China Medical University, Shenyang, 110042, China

2.Department of Ultrasound in Medicine, The First Affiliated Hospital of Ningbo University, Ningbo, 315201, China

3. Department of Radiology, The First Hospital of China Medical University,110001 Shenyang, China

#Those authors contributed equal to this work

*Corresponding author: Haiwei Li ([lihaiwei_2475@163.com](mailto:lihaiwei_2475@163.com))

**Methods**

**1. Materials**

The materials were all purchased from Tansoole, the reagent kits were obtained from Solarbio, and the antibodies were purchased from Biolegend. Nude mice (5 weeks, around 16g) were purchased from Peking University.

**2. Characterization**

Transmission electron microscopy (TEM) images, energy dispersive X-ray spectroscopy (EDS) and corresponding elemental mapping, High-resolution TEM (HRTEM) images, and selected area electron diffraction (SAED) patterns were applied to observe the particle size, morphology, element distribution, and crystal structure of HMC by using transmission electron microscopy (Talos F200x, USA). The crystal information of materials was assessed by X-ray diffractometer (XRD, D8 ADVANCE, BRUKER AXS GMBH, Karlsruhe, Germany), Dynamic Light Scattering (DLS, Zetasizer Advance ZSU3305, Malvern Instruments Limited, UK) was applied to measure the average hydrated particle size and zeta potential of materials. The absorption spectrum of TBNs was obtained through UV–vis–NIR spectrophotometric (Cary5000, Agilent, California, UK). The chemical composition and element status of materials were analyzed by X-ray photoelectron spectroscopy (XPS, AXIS Ultra DLD, Kratos, UK).

**3. Measurement of ROS generation**

The singlet oxygen (^1^O_2_) generation ability of HMC was assessed using 5,5-dimethyl-1-pyrroline-N-oxide (DMPO) as indicators on Electron paramagnetic resonance spectrometer (ESR, EMX nano D-76287, Bruker, Germany). For normoxic ROS generation tests, 1 mL sample dispersions (HMC, 200 μg mL^−1^) and 20 μL spin indicator molecule solutions (DMPO, TEMP, 1 mg mL^−1^) were mixed in sealed vials, and then exposed to US irradiation (1 MHz, 1 W cm^−2^) for 5 min.

**4. Sonodynamic performance of TBNs**

The singlet oxygen (^1^O_2_) generation ability of the sample was investigated using 1,3-Diphenylisobenzofuran (DPBF) measurements performed on an ultraviolet–visible–near-infrared (UV–vis–NIR) spectrophotometer. DPBF was dissolved into a 1 mL sample solution (HMC, 200 μg mL^−1^) in a glass cuvette. The ultrasound irradiation was applied to the system for 3 min at a power density of 1.0 W cm^−2^, the ultrasound was then shut off and UV measurements were conducted. After that, the ultrasound was turned on to irradiate the sample solution for another 3 min. The cycle was performed for 12 min and the absorption spectrum was recorded and compared from 300 nm to 500 nm.

**5. Cell cytotoxicity assays**

In order to detect the cell cytotoxicity of HMC for PAN02. Cells were initially seeded in 96-well plates. Subsequently, HMC varying concentrations (1, 2, 5, 10, 20 μg/mL) were introduced to each well and co-incubated for 24 hours. CCK-8 solution (10 μl) was added and incubated for an additional 2 hours at 37°C. The absorbance intensity of each well was measured using a microplate reader (iMark 168-1130, Biorad, U.S.) at 450 nm.

For the Live/Dead staining assay, PAN02 cells were seeded in 12-well plates and cultured overnight. Subsequently, HMC were added and incubated for 8 hours before 5 minutes of ultrasound (US) irradiation. After 16 hours, the treated cells were stained with a Calcein AM/PI double staining kit and observed via fluorescence microscopy.

**6. Western Immunoblotting**

PAN02 cell-derived proteins were extracted using lysis buffer supplemented with protease inhibitors. An equivalent amount of protein was resolved on a 12% SDS-PAGE gel and subsequently transferred onto a PVDF membrane. After blocking the membrane in 5% non-fat milk dissolved in TBST for 1 hour, it was incubated overnight at 4 ℃ with primary antibodies targeting specific proteins, including Bcl-2, C-caspase3, h2x.x-s139, Survivin and β-actin. Following three washes with Tris-Buffered Saline with Tween 20 (TBST), the membrane underwent a 1-hour incubation with corresponding secondary antibodies at room temperature, followed by additional TBST washes. Ultimately, the protein bands were detected using an enhanced chemiluminescence detection system.

**7. Detection of immunogenic cell death of 4T1 cells**

In the first step, the treated PAN02 cells were subjected to incubation with FITC-conjugated-CRT antibody, and the CRT levels were quantified using both flow cytometry and CLSM assays. For the second step, the treated cells were harvested, fixed with 4% paraformaldehyde, permeabilized with 0.1% Triton X-100, blocked with 10% BSA, and then exposed to anti-HMGB1 antibody. This was followed by staining with FITC-conjugated secondary antibody and DAPI. The subsequent measurement of HMGB1 levels was conducted through CLSM assays.

**8. *In vivo* Antitumor Immunotherapy.**

All animal experiments were carried out after approval by the ethical committee for animal care of Beijing Institute of Technology (Permit No. SYXK Jing 2017-0031).

A PAN02-tumor mouse model was established by subcutaneously injecting PAN02 cells into the back of female C56BL/6 mice. Subsequently, the mice were randomly divided into five groups: G1: PBS; G2:US; G3:PD-L1-IN-1@HMC (injected with PD-L1-IN-1@HMC through the tail vein); G4: HMC+US (injected with HMC through the tail vein and subjected to ultrasound treatment at the tumor site after 18 hours); G5: PD-L1-IN-1@HMC+US (injected with PD-L1-IN-1@HMC through the tail vein and subjected to ultrasound treatment at the tumor site after 18 hours). Body weights and tumor volumes were regularly monitored and recorded every two days for a total of 14 days. Tumor volume was calculated using the formula: tumor volume = 0.5 × length × width^2.

**9. Orthotopic tumor model and therapy**

Female nude mice were used for all animal studies, which were Mice were anesthetized and a 1 cm incision in the upper left abdominal quadrant was made. Te spleen and tail of the pancreas were then exposed, and 50 μL of PAN02 cells labeled with firefly luciferase suspended in PBS and Matrigel (phenol red-free, 2:3) were injected into the tail of the pancreas using a 0.3 mm needle. The spleen and pancreas were then restored to their appropriate positions within the abdomen, and the peritoneum was sutured using 4–0 absorbable sutures, after which the skin was closed with 6–0 non-absorbable sutures. Animals were then placed on a warming blanket until fully recovered from anesthetization. Subsequently, the mice were randomly divided into five groups: G1: PBS; G2:US; G3: HMC+US (injected with HMC through the tail vein and subjected to ultrasound treatment at the tumor site after 18 hours); G4: PD-L1-IN-1@HMC+US (injected with PD-L1-IN-1@HMC through the tail vein and subjected to ultrasound treatment at the tumor site after 18 hours). Tumor fluorescence intensity were regularly monitored and recorded every three days for a total of 12 days.

**10. Assessment of different groups of immune cells**

To assess the immune response induced by different therapy, various tissues, including the spleen, tumor-draining lymph nodes, primary tumor, distal tumor, and recurrent tumor, were surgically excised from mice across different experimental groups. Following digestion with collagenase IV (0.3 mg/mL) at 37°C for 1 h, single-cell suspensions were obtained through filtration using a 70 μm mesh. Subsequently, the harvested cells underwent CD16/CD32 antibody blocking for 15 min, followed by staining with eBioscience™ Fixable Viability Dye eFluor™ 506 for 15 min at 4℃.

The collected cells were then incubated with anti-CD45, anti-CD3, and anti-CD8 antibodies for evaluating the CD8^+^ T cell content within the tumors, employing standard flow cytometry protocols. Additionally, the cells were stained with anti-CD45, anti-CD3, anti-CD4, and anti-FoxP3 antibodies to assess the proportion of Treg T cells. For evaluating the proportion of NK cells, the collected cells were incubated with anti-CD45, anti-CD3, and anti-NK1.1+ antibodies. The frequency of mature DCs in lymph nodes was examined by flow cytometry after staining with anti-CD45, anti-MHCII, anti-CD11b, anti-CD80, and anti-CD86.

**11. Histological analysis**

Major organs, including the liver, spleen, kidney, heart, and lungs, along with tumor tissues from mice in each treatment group, were systematically harvested and preserved in 4% paraformaldehyde. Prior to electron microscopy, tissue samples underwent hematoxylin and eosin (H&E), terminal deoxynucleotidyl transferase dUTP nick end labeling (TUNEL), and Ki67 staining for detailed histological examination.


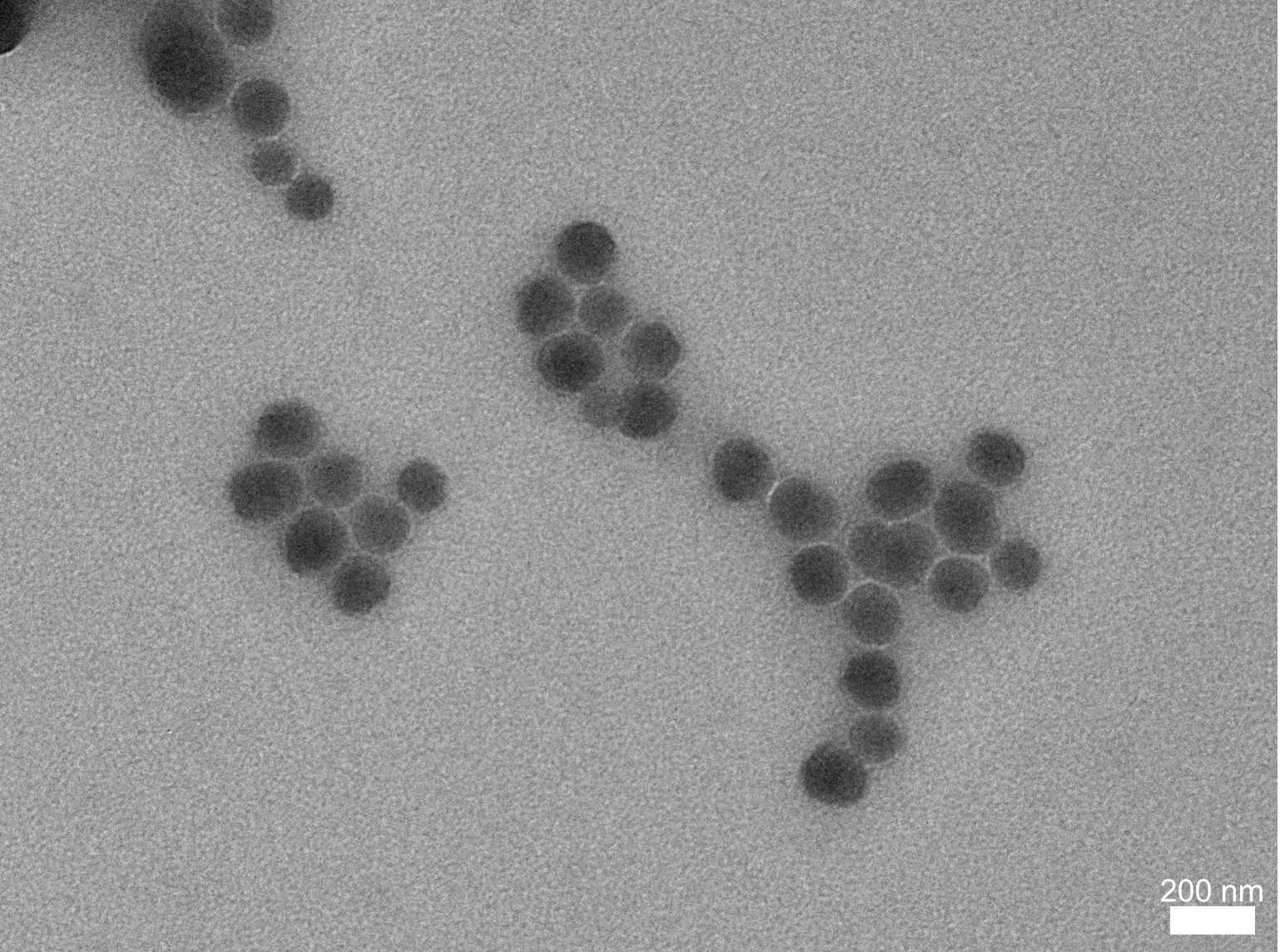


Figure S1. TEM result of SiO_2_@ZIF-8.

Figure S2. XRD pattern result of SiO_2_@ZIF-8.


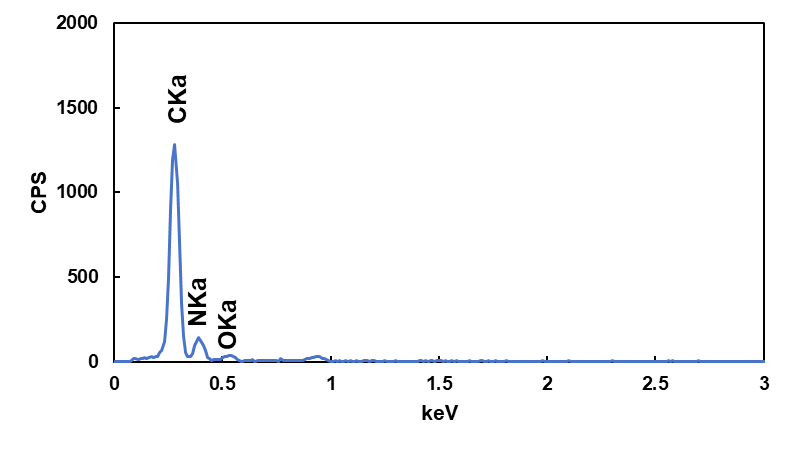


Figure S3. Elemental analysis result of HMC.


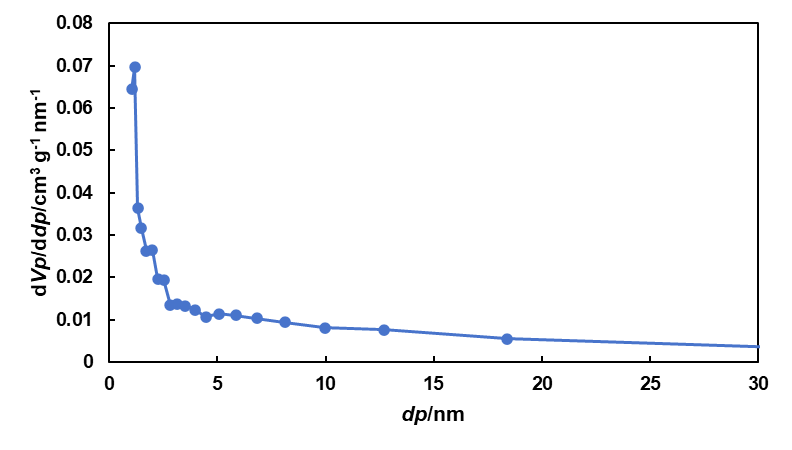


Figure S4. Pore size distribution of HMC.


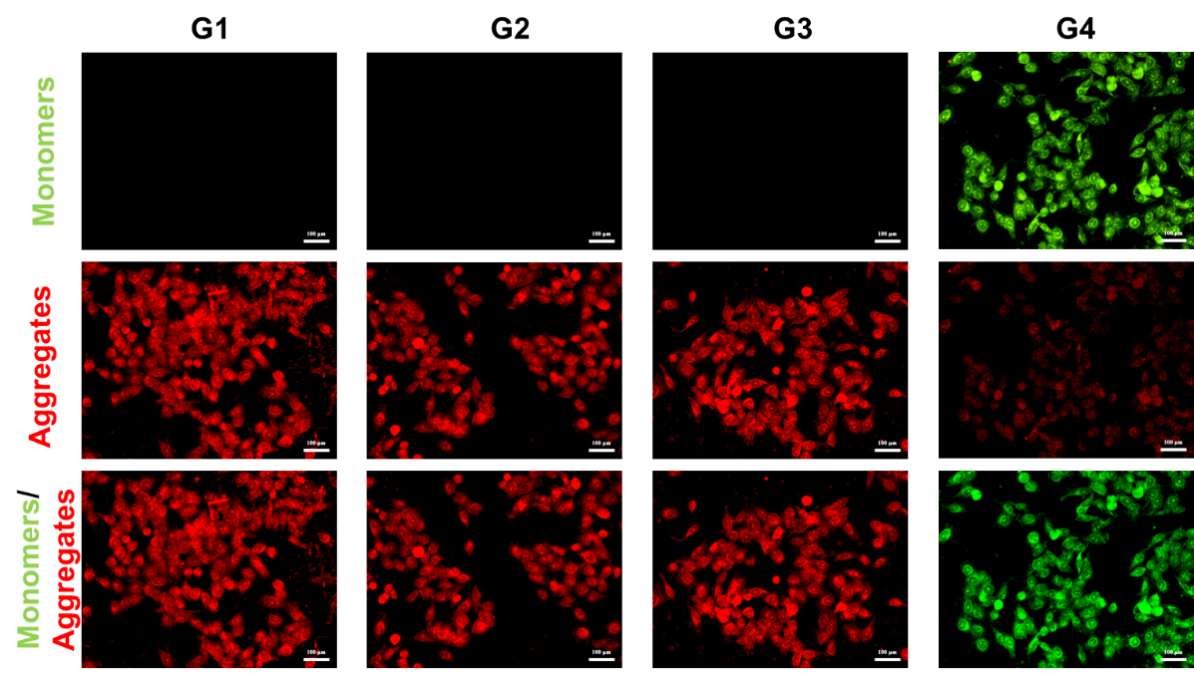


Figure S5. Fluorescence images of JC-1-labeled PAN02 cells after different treatments, Scale bar: 100 µm


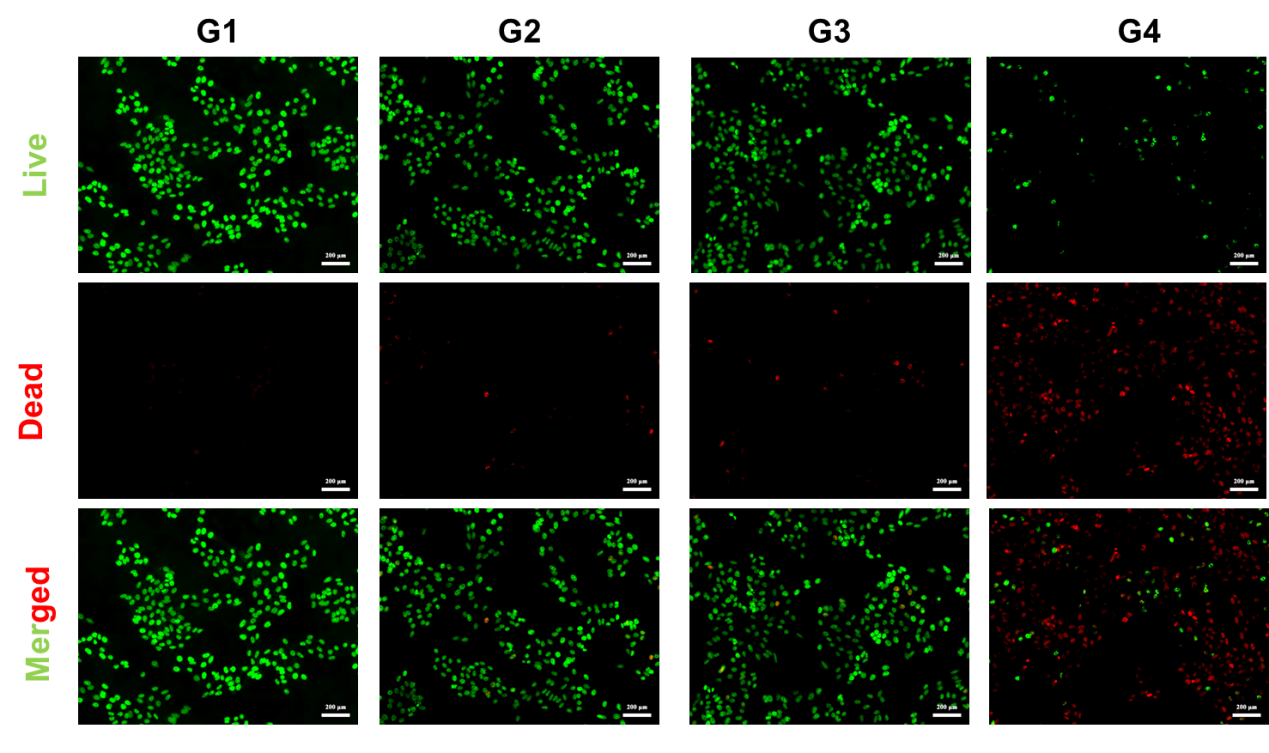


Figure S6. Representative images of calcein-AM/PI stained PAN02 cells after different treatments, Scale bar: 100 µm.


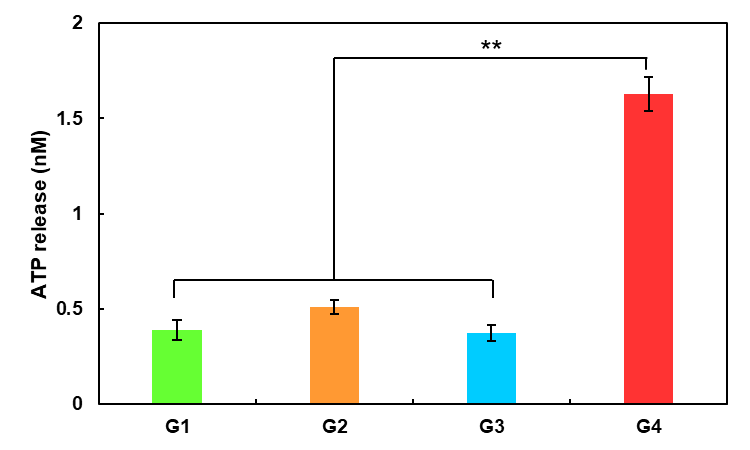


Figure S7. ATP release of PAN02 cells after different treatments. (n = 3, Mean ± SD)


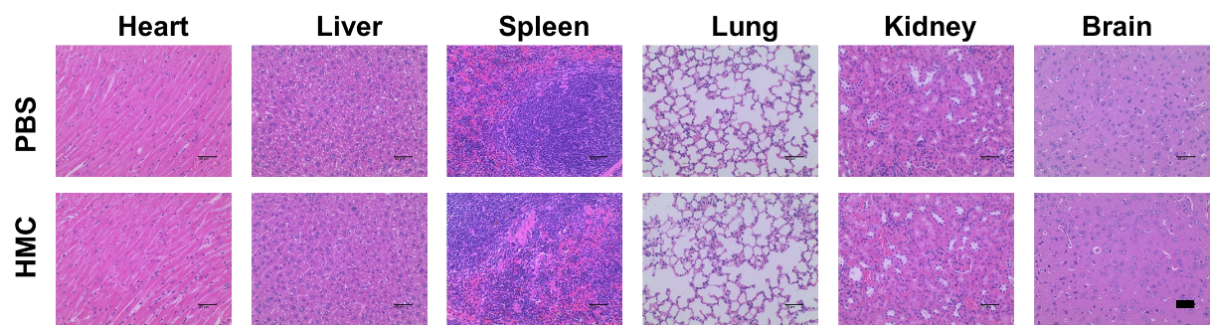


Figure S8. H&E staining of major organs of mice treated by PBS or HMC (50 mg/kg) after 14 days. Scale bar: 50 µm.


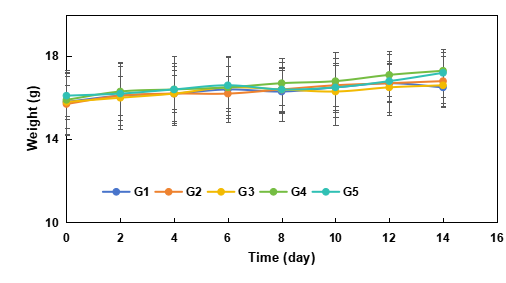


Figure S9. The weight change of mice under different therapy for 14 days. (n = 5, Mean ± SD).


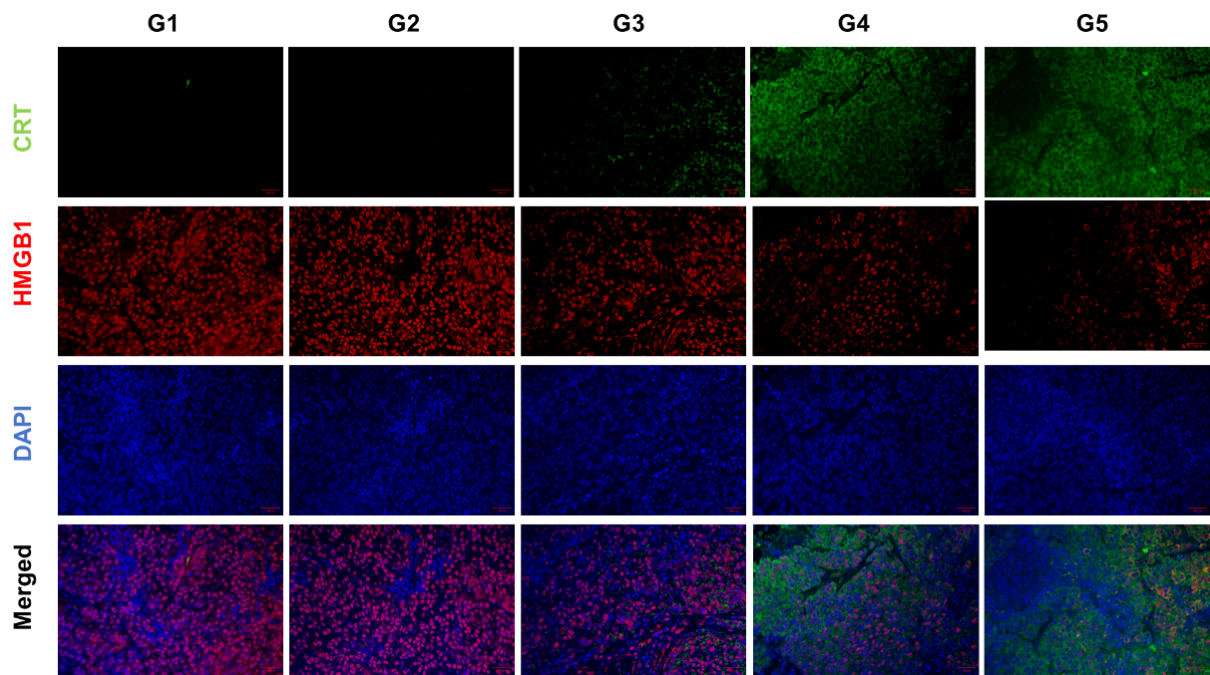


Figure S10. CRT and HMGB1G expression at the tumor site in PAN02 tumor-bearing mice after different treatments, Scale bar: 20 µm.


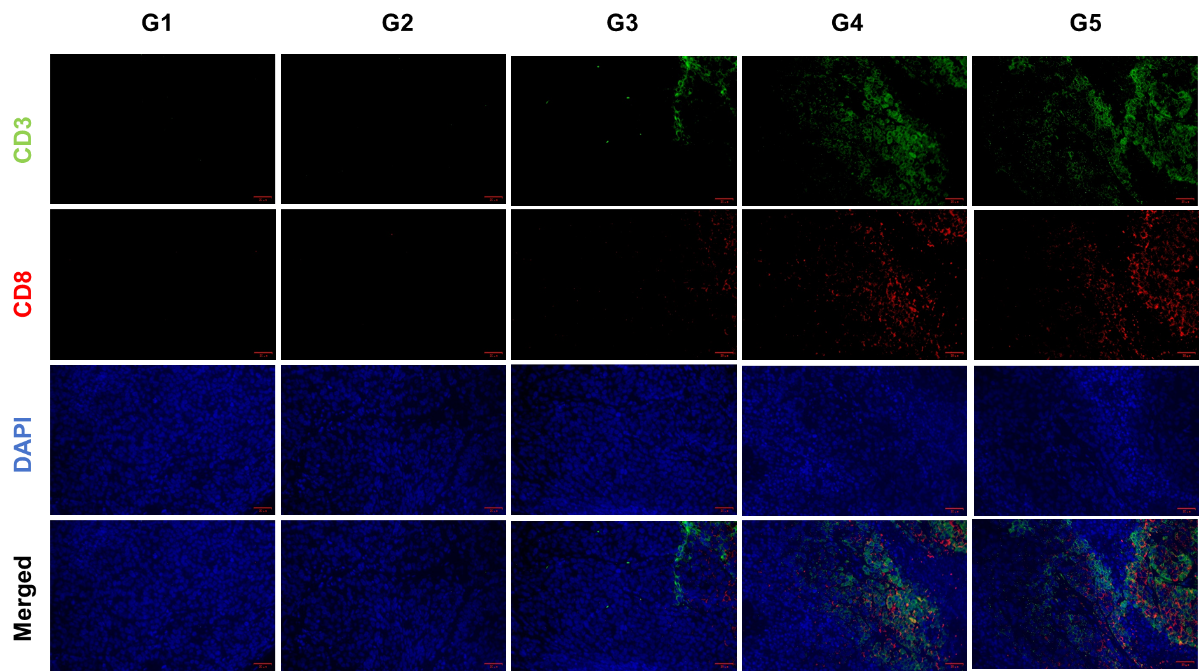


Figure S11. CD3/CD8 expression at the tumor site in PAN02 tumor-bearing mice after different treatments, Scale bar: 20 µm.


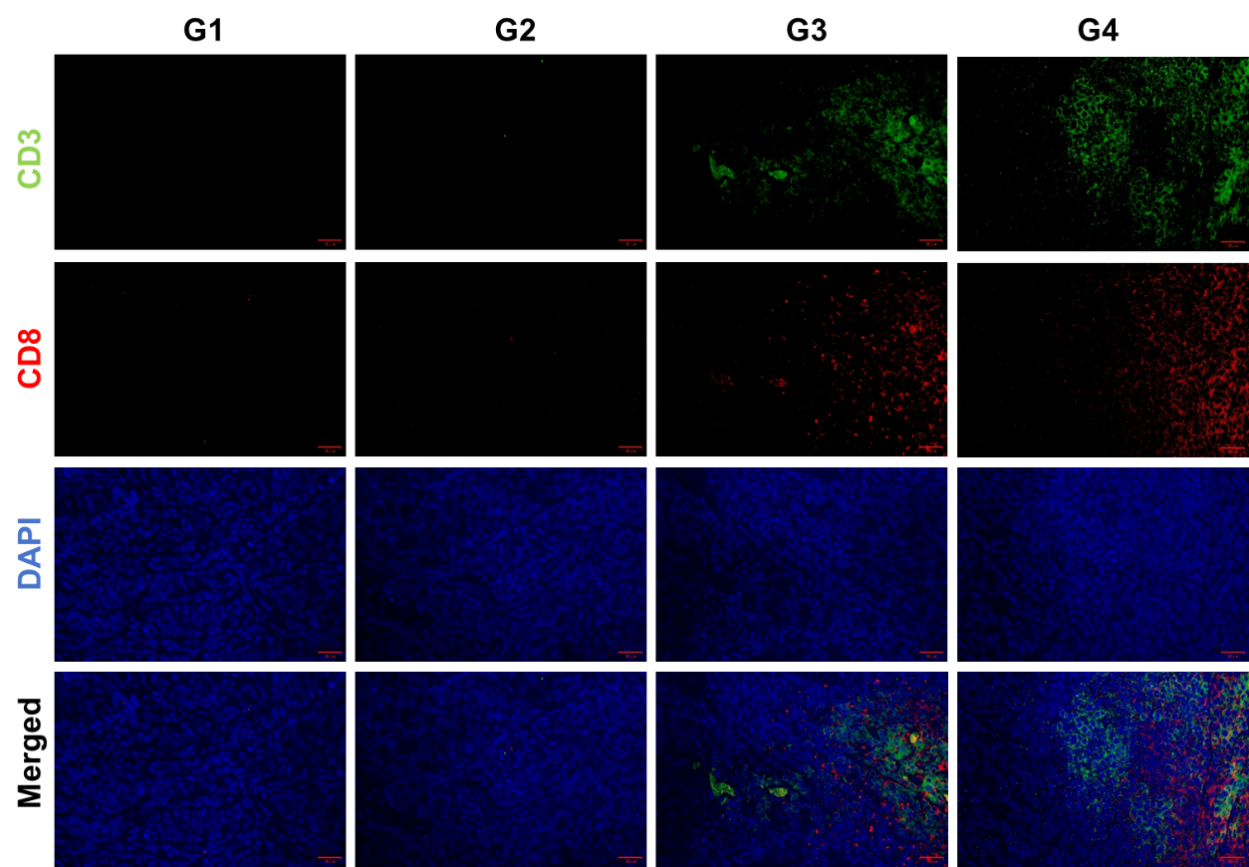


Figure S12. CD3/CD8 immunofluorescence staining of pancreatic tumor tissues from mice after 12 days of different treatments, Scale bar: 20 µm.


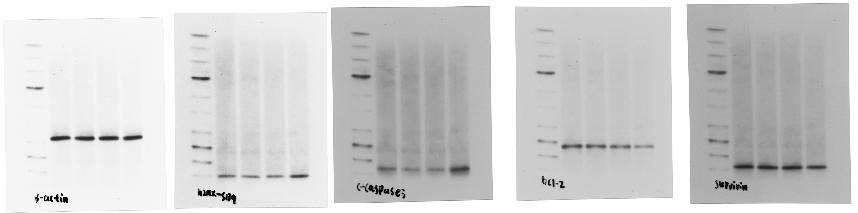


Figure S13. Western blot analysis results of Bcl-2, C-caspase3, h2x.x-s139, Survivin and β-actin.

Table S1. Quantitative analysis result of figure 5f

| Relative expression of Proteins | Bcl-2 | C-caspase3 | γH2AX-S139 | Survivin |
| --- | --- | --- | --- | --- |
| PBS | 0.345 | 0.152 | 0.248 | 1.268 |
| US | 0.295 | 0.099 | 0.211 | 1.272 |
| HMC | 0.267 | 0.107 | 0.209 | 1.371 |
| HMC+US | 0.154 | 0.249 | 0.381 | 0.947 |
